# LRP5 high bone mass (Worth-type autosomal dominant endosteal hyperostosis): case report and historical review of the literature

**DOI:** 10.1007/s11657-023-01319-6

**Published:** 2023-09-02

**Authors:** Giammarco De Mattia, Michele Maffi, Marta Mosca, Maurizio Mazzantini

**Affiliations:** 1 Rare Bone Diseases Clinic, Rheumatology Unit, Department of Clinical and Experimental Medicine, University of Pisa, Pisa, Italy; 2 Rheumatology Unit, Department of Clinical and Experimental Medicine, University of Pisa, Via Roma, 67 –, 56126 Pisa, Italy

**Keywords:** Worth disease, Autosomal dominant osteosclerosis, High bone mass, LRP5 HBM, Endosteal hyperostosis

## Abstract

**Purpose:**

LRP5 high bone mass (HBM) is an autosomal dominant endosteal hyperostosis caused by mutations of the *low-density lipoprotein receptor-related protein 5* (*LRP5*) gene. Alternative names included “autosomal dominant osteosclerosis” and “Worth disease.” The aim of the paper is to provide an historical overview of a disorder whose literature is complicated and confusing due to the past use of several denominations and lack of reviews.

**Methods:**

We collected case reports of HBM with evidence of autosomal dominant transmission preceding the identification of the LRP5 mutations in 2002 (Worth-type endosteal hyperostosis) and cases of LRP5 HBM confirmed by genetic analysis since 2002. The prevalence of relevant clinical and laboratory findings was estimated. We described an affected woman with neurological manifestations.

**Results:**

A 44-year-old Caucasian woman with torus palatinus complained of headache, hypo-/anosmia, and complete mixed deafness. Dual-energy X-ray absorptiometry (*DEXA*) scan revealed elevated bone mass. The A242T mutation of the *LRP5* gene was detected. Including the present case, 155 patients have been reported to date. Neurological involvement and increased serum alkaline phosphatase (ALP) were present in 19.4% and 3.7% of cases, respectively. Facial changes and torus palatinus were observed in 61% and 41% of cases, respectively.

**Conclusions:**

We present the only historical review on Worth-type endosteal hyperostosis, now known as LRP5 HBM. Neurological manifestations, previously considered absent in the disease, affect 19.4% of the patients. Genetic analysis and appropriate denomination of LRP5 HBM are fundamental for diagnosis and to mitigate the confusion that has long characterized this disease.

## Introduction

Endosteal hyperostoses are a group of rare disorders characterized by elevated bone mass [1]. They have been classified into two autosomal recessive forms caused by mutations in the *SOST* gene coding for sclerostin—Van Buchem’s disease (OMIM #239100) and sclerosteosis (OMIM #269500)—and an autosomal dominant high bone mass (HBM) phenotype [2], now known to comprise two disorders called LRP5 HBM (OMIM # 144750) and LRP6 HBM [3]. This autosomal dominant HBM phenotype, described for the first time by Worth and Wollin in 1966 [4] and hence sometimes historically called with the eponym “Worth disease” or “Worth-type endosteal hyperostosis,” has been initially described as a milder form of hyperostosis characterized by torus palatinus, normal levels of serum ALP, and absence of neurological manifestations [5–7] until the first report of neurological involvement in 1976 [8]. On the other hand, Van Buchem’s disease and sclerosteosis feature elevated levels of ALP in more than half of the cases and a higher rate of neurological complications [9–15], with sclerosteosis also being characterized by syndactyly of the second and third fingers and nail dysplasia [16]. Over the years, several denominations have been employed in the literature to refer to autosomal dominant HBM phenotype besides “Worth disease” [5–7], including “autosomal dominant osteosclerosis” [17–22], “autosomal dominant osteopetrosis, type 1” [23], and “Van Buchem’s disease, type 2” [24]. The most recent one is “LRP5 HBM” [3], a term introduced after the discovery of the underlying pathogenic role of missense mutations in the *low-density lipoprotein receptor-related protein 5* (*LRP5*) gene in 2002 by Little et al. [25]. Furthermore, the detection of pathogenic mutations in the *low-density lipoprotein receptor-related protein 6* (*LRP6*) gene in some patients with autosomal dominant HBM phenotype led to the description of a clinically identical disorder in 2019, called LRP6 HBM [3]. Importantly, the identification of these mutations has facilitated the differential diagnosis of LRP5 HBM with other disorders such as osteopetrosis, progressive diaphyseal dysplasia (Camurati-Engelmann disease), Paget’s disease of the bone, and pycnodysostosis, all of which are often characterized by localized lesions [26]. Nevertheless, the past use of several different names for autosomal dominant HBM phenotype may render the literature complicated and confusing. In this paper, we describe a woman with HBM with neurological involvement and carrying a *LRP5* mutation. For the first time, we provide a comprehensive historical review of the literature after having gathered all case reports of this condition, with the aim to establish the prevalence of the main clinical, genetic, and laboratory findings.

## Methods

A comprehensive review of the literature on LRP5 HBM was carried out. Medline databases (PubMed) were consulted using the following keywords: “Worth disease,” “Worth syndrome,” “Autosomal dominant osteosclerosis,” “Autosomal dominant endosteal hyperostosis,” “LRP5 high bone mass,” and “autosomal dominant hyperostosis corticalis generalisata.” As previously mentioned, the pathogenic role of mutations in the *LRP5* gene was described for the first time in 2002 [25]. Thus, for the timespan between 1966 and 2001, we included all case reports describing patients with HBM phenotype with evidence of autosomal dominant transmission and without clinical and radiologic signs of other metabolic bone diseases, as confirmatory genetic analysis was not available before 2002. On the other hand, case reports written in 2002 or later were included only when *LRP5* mutations were detected and mentioned. Throughout this review, the case reports in the literature with the diagnoses mentioned above are labeled with the historical term “Worth-type endosteal hyperostosis” for cases reported before 2002 and with the term “LRP5 HBM” for cases described after 2002. This review does not include patients affected by LRP6 HBM. We performed a comprehensive clinical, radiological, and genetic assessment of a woman affected by LRP5 HBM with neurological involvement.

## Case report

**Fig. 1:**
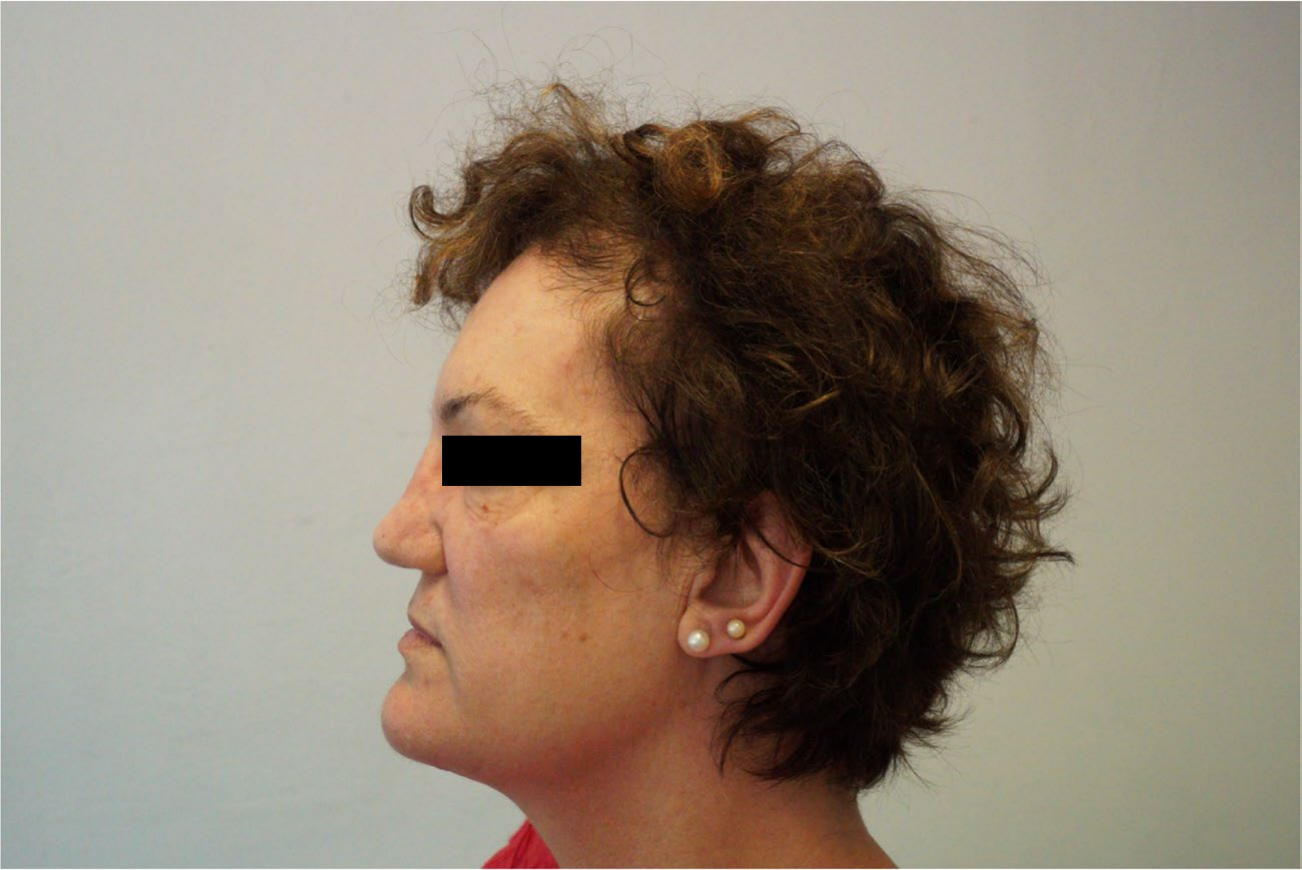
Clinical examination of our patient revealed flat nasal bridge, frontal bossing, and deepened and widened jaw with increased gonial angle. Written informed consent was obtained from the patient for publication of the photograph

**Fig. 2:**
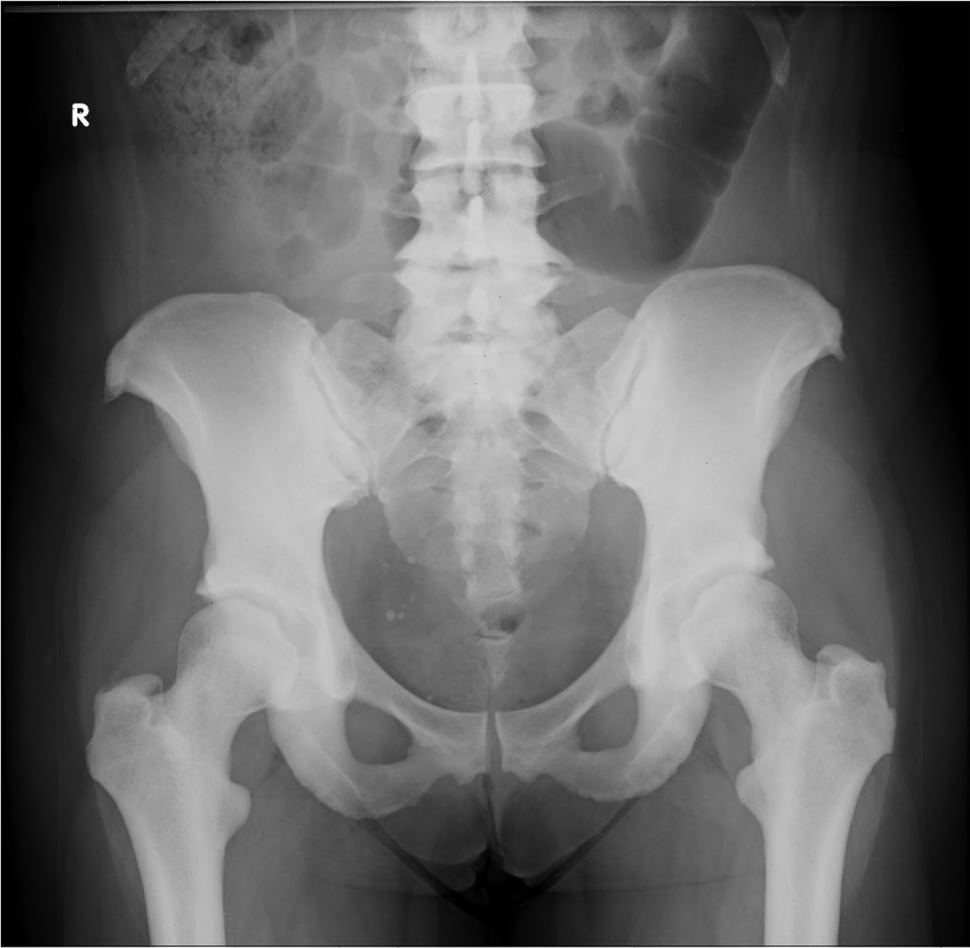
X-rays showing endosteal hyperostosis of femurs and osteosclerosis of hip and vertebrae. Written informed consent was obtained from the patient for publication of the radiograph

**Fig. 3:**
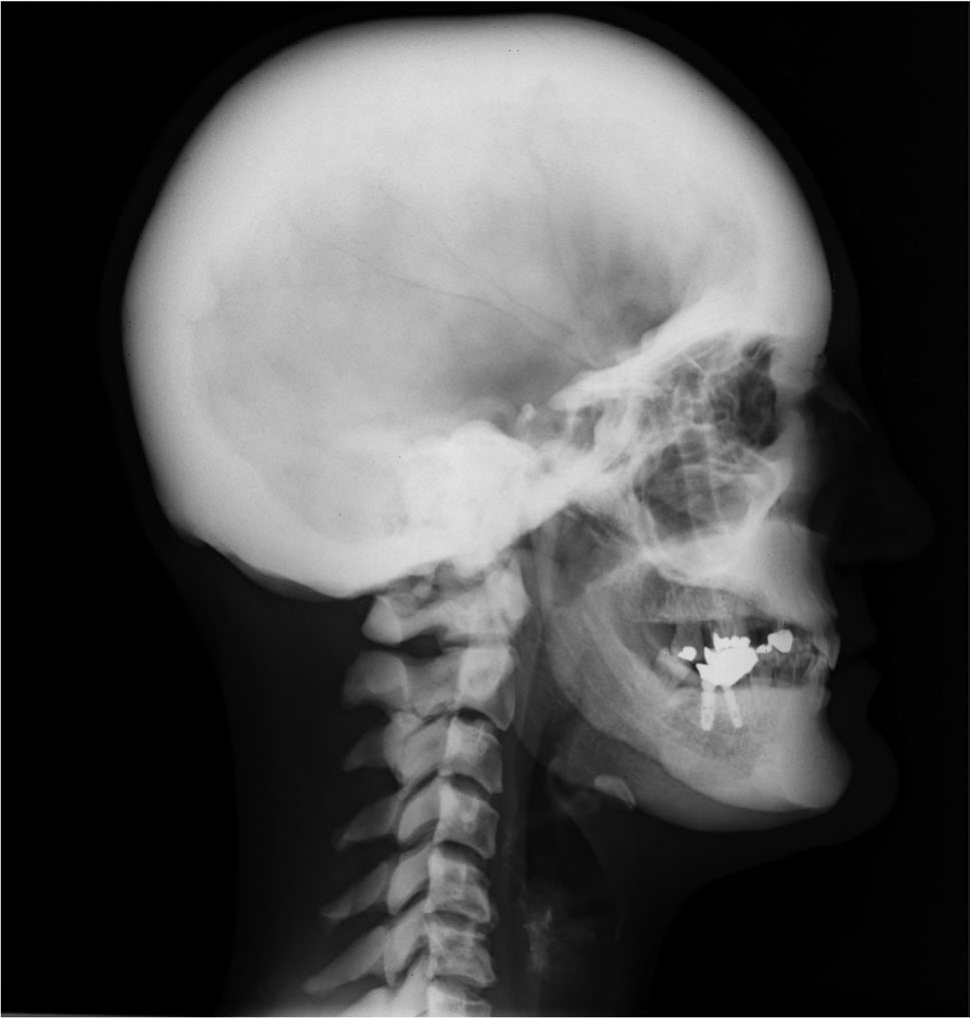
X-rays showing severe osteosclerosis of the skull, particularly at the base. Written informed consent was obtained from the patient for publication of the radiograph7

A 44-year-old Caucasian woman came to the attention of our Rheumatology Unit in 2009 for evaluation of her chronic and debilitating lumbodorsal pain (Visual Analog Scale 8/10) which had recently caused her to quit her job as a cook. She was 166 cm (65.5 inches) in height, 80 kg (176 lb) in weight, and had normal intelligence. She reported that her low back pain started at age 12 and has been persistent thereafter. Lumbosacral spine X-rays performed at that time revealed “marble bones” for which she was diagnosed with “osteopetrosis” and unsuccessfully treated with non-steroidal anti-inflammatory drugs (NSAIDs) and physical therapy. Pain exacerbated with time and later also involved the cervical spine, elbows, wrists, and shoulders. At age 20, the patient reported lower limb paresthesias and recurrent episodes of nephrolithiasis and frontoparietal headache without nausea or vomiting. In the same period, she noticed the appearance of coarse “facial changes” that became more evident with time, as illustrated in photographs brought to our attention. Between ages 25 and 30, the patient suffered from sinusitis, recurrent bilateral trigeminal neuralgia, bilateral carpal tunnel syndrome, dysgeusia and hypo-/anosmia, oral cavity paresthesias, arthralgias of upper and lower limbs, and self-limiting episodes of unilateral facial palsy. At age 35, external hearing aids were placed due to complete, bilateral, and mixed hearing loss caused by the narrowing of internal and external acoustic meati. Previous CT scans of the petrous bone showed bilateral exostoses in the inner ear, whereas the vestibular system and the semicircular canals were normal. At presentation, clinical examination revealed a deepened and widened jaw with increased gonial angle, frontal bossing, flat nasal bridge, hypertelorism, and torus palatinus (Fig. 1). There were no syndactyly or evident deformations of extracranial bones. Laboratory findings were normal, including complete blood count (CBC) and serum hemoglobin 13.4 g/dL (reference values: 11.5–15), calcium 9.9 mg/dL (8.6–10.2), phosphorus 3.2 mg/dL (2.7–4.5), ALP 54 IU/L (35–105), 25(OH)D 12.8 ng/mL (11–70), osteoprotegerin 2.3 pmol/L (1.4–6), RANKL 0.02 pmol/L (<6), PTH 49 pg/mL (13–54), C-reactive protein 0.1 mg/dL (<0.5), P1NP 68.4 ng/mL (15.1–73.9), C-terminal telopeptide (CTX) 0.220 ng/mL (<0.6), and sclerostin 1.3 ng/mL (0.5–2.4). The patient underwent a complete skeletal survey which revealed endosteal hyperostosis and severe osteosclerosis affecting clavicles, ribs, vertebrae, hip (Fig. 2), limbs, and cranium (Fig. 3), in the absence of deformities except for the square jaw. She denied previous bone fractures and reported inability to float in water. Bone scintigraphy with technetium-99 m did not show any abnormal accumulations. Dual-energy X-ray absorptiometry (*DEXA*) scan (GE Medical Systems, Lunar) revealed *Z*-score +11.1 (*T*-score +10.8) and bone mineral density (BMD) of 2.5 g/cm^2^ for L1-L4 spine, *Z*-score +6.2 (*T*-score +6.5), and BMD of 1.8 g/cm^2^ for femoral neck, and *Z*-score +8.1 (T-score +8.5) and BMD of 2 g/cm^2^ for total hip. The patient never suffered from vision disturbances, and visual field testing was normal. However, the ophthalmologist found bilateral papilledema, which prompted further testing for intracranial hypertension. Head CT scan and MRI revealed normal ventricular system but detected mild cerebellar tonsillar herniation through the foramen magnum. Neither pyramidal tract signs nor cerebellar disturbances were present. Genetic analysis through Sanger-sequencing detected the heterozygous missense mutation A242T in exon 4 of the *LRP5* gene coding for the first β-propeller domain of the LRP5 protein and showed a G/A transition in position 51145 of DNA. The patient reported that none of her relatives ever had facial deformities or radiologic findings suggesting hyperostosis. The patient’s mother and father died at age 61 and 70, respectively, both from ischemic heart attack; none of them apparently ever performed *DEXA* scan. Her 47-year-old sister and her two adolescent children had normal *DEXA* scans and were in good health. Her 40-year-old brother suffered from chronic low back pain but refused to undergo any investigation. In 2013, hysteroannessiectomy was performed due to leiomyomas, thereby inducing iatrogenic menopause. At the latest evaluation in May 2022, the patient reported diffuse joint pain compatible with osteoarthritis, sleep disturbances with fibromyalgia-like symptoms, recurrent frontoparietal headaches, and numbness of oral cavity and limbs. *DEXA* scan performed in the same period revealed *Z*-score +11.0 (*T*-score +10.4) and BMD of 2.4 g/cm^2^ for lumbar spine, *Z*-score +5.1 (*T*-score +4.4) and BMD of 1.5 g/cm^2^ for femoral neck, and *Z*-score +6.9 (*T*-score +6.4) and BMD of 1.8 g/cm^2^ for total hip.

## Review of the literature

In 1955, Van Buchem et al. introduced the term “hyperostosis corticalis generalisata familiaris” to describe a condition characterized by a generalized increase in bone density on X-rays. The authors observed bilateral thickening of the diaphyseal cortex of long and short tubular bones and osteosclerosis of the skull, ribs, pelvis, vertebrae, and clavicles. Metaphyses and epiphyses of long bones were spared. This disorder, thereafter called Van Buchem’s disease, was transmitted with an autosomal recessive pattern and was frequently characterized by elevated serum ALP and neurological involvement, including cranial nerve deficits due to encroachment on the foramina of the skull base [11].

In 1966, Worth and Wollin described nine related Canadian individuals affected by skeletal radiographic changes resembling those described by Van Buchem. However, their series was characterized by normal levels of serum ALP and absence of neurological involvement. Furthermore, the pedigree was suggestive for an autosomal dominant pattern of inheritance, and a bony outgrowth of the hard palate (torus palatinus) was present in three cases [4], whereas it was not noted in the cases described by Van Buchem. A Japanese series of six patients resembled these findings, except for the absence of torus palatinus [5]. In 1971, Maroteaux et al. published three cases of endosteal hyperostosis under the term of “Worth-type hyperostosis corticalis generalisata”; findings included torus palatinus, normal serum ALP, and the absence of neurological involvement in all patients. The pedigree of their series suggested an autosomal dominant transmission of the disorder [6]. In 1976, Beals speculated for the first time the existence of a milder and more benign Worth-type autosomal dominant disease as opposed to the more severe and autosomal recessive Van Buchem’s disease [7]. In 1977, Gorlin and Glass introduced the term “autosomal dominant osteosclerosis” to separate this apparently milder entity from Van Buchem’s disease [17]; this denomination has been employed in a few subsequent publications [18–22]. Other names include “autosomal dominant osteopetrosis, type 1” [23], “Van Buchem’s disease, type 2” [24], and more recently “LRP5 high bone mass (HBM)” [3].

The diagnosis of LRP5 HBM in patients with HBM detected by *DEXA* scan is nowadays possible thanks to genetic analysis of the *LRP5* gene. However, mutations in this gene were identified as the cause of this disorder only in 2002 [25]. This review includes all cases of HBM phenotype with evidence of autosomal dominant transmission—and without other signs of other metabolic bone diseases—described before 2002, starting from Worth and Wollin’s publication in 1966. On the other hand, we included papers written in 2002 or later only if *LRP5* gene analysis was performed. According to these parameters, we included 155 patients (74 males and 81 females) described in 32 case reports published in the literature [4–8, 16, 18–22, 24, 25, 27–42, 42–44], including our patient herein described. Sixty-four patients (41%) out of these 155 underwent genetic analysis of the *LRP5* gene and can be considered as being affected by LRP5 HBM. The main features characterizing each series are reported in Table 1.

**Table 1:** The main characteristics of the 155 patients included in the review are reported. Elevated levels of serum ALP were present in 5 cases. Facial changes and torus palatinus were observed in 93 and 58 patients, respectively. Cranial nerve deficits and/or other neurologic complications (e.g., intracranial hypertension, headache, spinal canal stenosis, sensitive neuropathy, papilledema, type I Chiari malformation, nausea and/or vomiting, migraine, and cerebellar tonsillar herniation) were present in 30 patients. M, males; F, females; ALP, alkaline phosphatase; LRP5, low-density lipoprotein receptor-related protein 5; NA, not applicable. ^a^In 2002, Little et al. [25] detected the LRP5 mutation in the pedigree which had been described by Johnson et al. in 1997 [32]. ^b^The paper by Kwee et al. [22] published in 2005 described the expanded pedigree of a family which had been originally described in a paper written in Dutch by Brouwer OH et al. in 1970. The authors performed genetic analysis of the *LRP5* gene and detected the same LRP5 mutation in 5 individuals; the other 8 individuals of the pedigree described in 1970 all had clinical and radiologic findings compatible with HBM

Case report	Year	Country	Number of cases	Age (years)	M	F	High serum ALP	Facial changes	Torus palatinus	Cranial nerve deficits	Other neurologic complications	*LRP5* mutation present
Worth and Wollin [4]	1966	Canada	9	13–82	7	2	0	4/9	3/9	0	0	NA
Russell et al. [5]	1968	Japan	6	15–49	1	5	0	5/6	0	0	0	NA
Maroteaux et al. [6]	1971	France	3	8–69	1	2	0	0	3/3	0	0	NA
Segond et al. [27]	1973	France	3	27–61	2	1	0	0	0	0	0	NA
Beals [7]	1976	USA	14	64 Unknown for 13 cases	7	7	1/14	14/14	14/14	0	0	NA
Owen [41]	1976	UK	6	7–64	3	3	Unknown	6/6	Unknown	0	0	NA
Lapresle et al. [8]	1976	France	8	17–74	2	6	0	8/8	2/8	6/8	0	NA
Vayssairat et al. [28]	1976	France	5	14–52	4	1	0	0	0	0	0	NA
Gelman [18]	1977	USA	2	31–57	1	1	0	Unknown	Unknown	0	0	NA
Demonchy et al. [40]	1978	France	1	76	0	1	1/1	1/1	1/1	1/1	0	NA
Moretti et al. [29]	1982	Italy	8	5–49	3	5	0	0	0	1/8	1/8	NA
Yasuda et al. [19]	1986	Japan	8	18–58	5	3	0	0	1 Unknown in 7	0	2/8	NA
Perez Vincente et al. [30]	1987	Spain	2	28–51	1	1	1/2	2/2	0	1/2	1/2	NA
Irie et al. [16]	1989	Japan	1	35	1	0	Unknown	0	0	0	0	NA
Adès et al. [31]	1994	Australia	3	11–38	1	2	0	3/3	2/3	2/3	3/3	NA
Johnson et al. [32]	1997	USA	12	18–86	6	6	0	0	0	0	0	Yes (see Little et al. [25])^a^
Scopelliti et al. [24]	1999	Italy	1	25	0	1	1/1	1/1	0	1/1	1/1	NA
Curran et al. [20]	1999	UK	3	24–62	0	3	0	3/3	3/3	0	0	NA
Little et al. [25]	2002	USA	6 (added to cases of Johnson et al. 1997 [32])	Unknown	3	3	0	0	0	0	0	Yes
Boyden et al. [33]	2002	USA	7	41–77	5	2	0	7/7	7/7	0	0	Yes
Renton et al. [21]	2002	UK	1	20	0	1	0	1/1	1/1	0	0	Yes
MP Whyte et al. [43]	2004	USA	1	37	0	1	0	1/1	1/1	1/1	1/1	Yes
Boyden et al. [44]	2004	USA	10	10–83	4	6	Unknown	10/10	9/10	3/10	4/10	Yes
Rickels et al. [34]	2005	USA	1	59	0	1	0	1/1	1/1	0	0	Yes
Kwee et al. [22]	2005	The Netherlands	13	2–47	7	6	0	13/13	0	2/13	3/13	Yes (5/13)^b^
Balemans et al. [39]	2007	Belgium	1	55	0	1	0	0	1/1	0	0	Yes
Wang et al. [35]	2013	China	2	40–64	0	2	1/2	2/2	2/2	0	0	Yes
Gregson et al. [36]	2016	UK	11	21–76	6	5	0	8/11	4/11	3/11	0	Yes
Costantini et al. [37]	2017	Finland	1	57	1	0	0	0	0	0	1/1	Yes
Roetzer et al. [38]	2018	Austria	2	23–53	0	2	Unknown	0	0	1/2	1/2	Yes
Zhao et al. [42]	2023	China	3	22–50	3	0	0	2/3	2/3	1/3	1/3	Yes
Present paper	2023	Italy	1	44	0	1	0	1/1	1/1	1/1	1/1	Yes

The available literature shows that this rare bone disorder is characterized by radiographically evident and generalized endosteal hyperostosis causing cortical thickening of diaphyses of long bones and osteosclerosis of ribs, clavicles, vertebrae, hip, metacarpals, and calvaria with loss of the diploë [17]. Such high bone density does not interfere with hematopoiesis, as observed in our patient who did not feature anemia, or with bone outer shape and dimensions [32]. On this regard, this disorder is different from osteopetrosis, which is characterized by deformities attributable to defects in bone remodeling and often by anemia [45]. X-rays may show a considerable narrowing of the medullary canal of the long bones [27]. This condition may be discovered incidentally [41], and in many cases, it is asymptomatic [27]. The prognosis is good, with patients generally having a normal lifespan [46].

Bilateral broadening and prognathism of the mandible with increased gonial angle are often described [21] and may lead to malocclusion [46]. Other common craniofacial changes include frontal bossing, flat nasal bridge, abnormal bony outgrowths in the hard palate and mandible, called torus palatinus and mandibularis, respectively, and more rarely hypertelorism [31]. Facial changes usually start at puberty, are often evident by the end of adolescence, and cease with growth [21], like in our case. Facial changes were present in 93 cases and were not mentioned in 2 patients [18]. Thus, according to our review, they are present with a prevalence of approximately 61%.

Torus palatinus was noted in 58 individuals [4, 6–8, 19–21, 31, 36, 40], including our case, whereas it was not mentioned in 15 patients [18, 19, 41]. Therefore, the prevalence of torus palatinus out of the remaining 140 cases is approximately 41%.

Inability to float in water has been reported [31, 33, 36, 46], as in our case. Craniosynostosis, defined as the premature fusion of one or more of the cranial sutures, and mild developmental delay have been reported [22]. Nail hypoplasia and syndactyly have never been reported; instead, these clinical findings are present in sclerosteosis [31].

Although cranial nerve deficits are a common finding in sclerosteosis and are described in about half of the cases of Van Buchem’s disease [29], the absence of neurological involvement has long been considered one of the identifying aspects of Worth-type endosteal hyperostosis as mentioned in some of the oldest case reports [4–7, 17, 18, 27]. The first report of neurological involvement was published by Lapresle et al. in 1976, who described a young girl affected by left trigeminal neuralgia along with homolateral sensorineural hearing loss, visual field defects, and decreased corneal sensitivity, thereby suggesting unilateral deficits of the second, fifth (ophthalmic and maxillary divisions), seventh, and eight cranial nerves. The authors attributed these findings to unusually pronounced facial dysmorphisms [8]. Demonchy et al. reported a case presenting with progressive unilateral compression of the optic, trigeminal, and facial nerves [40]. Moretti et al. reported severe mixed hearing loss, partial visual field defects, and mild unilateral facial paralysis in one case who required decompressive craniotomy [29]. Two related Japanese patients presented with chronic occipital headache and spinal canal stenosis causing pain and paresthesias in the upper limb [19]. Spinal canal stenosis has been reported [37, 43]. In 1987, Perez-Vincente et al. described symptoms related to chronic intracranial hypertension and mild corticospinal tract abnormalities in one patient, and when reviewing the literature, they noted that 28% of the autosomal dominant cases published up to that time were somewhat affected by neurological complications as opposed to 81% in Van Buchem’s disease [30]. Adès et al. reported bilateral papilledema in a family of three patients; two patients also had a history of unilateral facial palsy, and one had narrow internal auditory meati and inferior herniation of cerebellar tonsils into the foramen magnum [31], like our case. In the kindred described by Kwee et al., one patient suffered from hearing loss, headache, and dizziness, two from headache only, and one from irreversible visual deficits due to optic nerve atrophy secondary to intracranial hypertension despite an urgent craniotomy had been performed [22]. A young Austrian patient had congenital hearing loss and headache with intermittent nausea and vomiting secondary to impaired cerebrospinal fluid (CSF) circulation, for which early cochlear implantation and surgical removal of part of her occipital bone were performed [38]. Type I Chiari malformation was described in two individuals with LRP5 HBM [43]. Our patient reported complete and bilateral hearing loss, trigeminal neuralgia, transient facial palsy, and hypo-/anosmia, thus indicating deficits of the first, fifth, seventh, and eight cranial nerves. To our knowledge, this is the first case of LRP5 HBM in which olfactory deficits have been reported. Furthermore, our patient was affected by early-onset headache, mild cerebellar tonsillar herniation, and bilateral papilledema. These findings are consistent with chronic intracranial hypertension, even though pyramidal tract signs or cerebellar disturbances were absent. In summary, neurological involvement may occur because of nerve tissue compression by hyperostotic bone, cerebellar disturbances due to a reduction in size of the posterior cranial fossa or tonsillar herniation, and chronic intracranial hypertension [30]. According to our review of the previous literature and considering the present case, cranial nerve deficits and/or other neurological complications have been reported in 30/155 cases, corresponding to a prevalence of 19.4%.

Laboratory findings are usually normal, as opposed to Van Buchem’s disease in which increased levels of serum ALP are frequently reported [17]. Most patients affected have normal serum ALP, including our case. Serum ALP has not been mentioned or investigated in 19 patients [16, 38, 41, 43]. Elevated levels of serum ALP have been reported in 5 [30, 35, 40] of the remaining 136 cases, corresponding to an overall prevalence of 3.7%.

In 2001, Beals et al. carried out a postmortem examination of a man whom he had described in 1976 [7] and who had willed his body for study following his death. They performed biomechanical testing to assess bone quality and measured ash weight to assess bone quantity. Cortical and cancellous bone from this patient were stiffer than normal bone. Histology revealed packed haversian systems with normal cellular architecture in cortical bone and normal but thick trabeculae in cancellous bone [46]. A biopsy of the acromion and of the right ilium in another report also demonstrated thickened but normal mature lamellar bone [18]. Similar findings have been reported in a more recent case [38]. These properties provide affected patients with a certain degree of protection from fractures [46]; indeed, only a 85-year-old man with a fracture of the femur [4], a 76-year-old man who had sustained two very high impact fractures of fibula and elbow 30 years earlier [36], and a 12-year-old boy with a limb fracture while skiing [16] have been reported, whereas all the remaining patients described in the literature never had a bone fracture.

No consensus on treatment is available. A few reports described surgical treatment of neurologic complications [29, 38] and of facial deformities [21]. In 2013, at age 48, our patient required surgery for leiomyomas and underwent hysteroannessiectomy. In our opinion, it is unlikely that iatrogenic menopause was responsible for the slight decrease in bone mass seen at *DEXA* scan in 2022 compared to 2009, since one study showed that murine models carrying *LRP5* gain-of-function mutations were somewhat protected from bone loss even in the presence of bone-wasting stimuli, such as ovariectomy [47].

### The role of LRP5 mutations in HBM

Bone mass is influenced by environmental factors, such as age, nutritional status, physical activity, and comorbidities, and by genetic factors [25]. In 1997, Johnson et al. mapped to chromosome 11q12-13 a genetic locus that determines an autosomal dominant trait of HBM [32]. In 2002, Little et al. discovered that a gain-of-function mutation causing a glycine-to-valine amino acid change (G171V) in the first β-propeller domain of the LRP5 protein encoded by the *low-density lipoprotein receptor-related protein 5* (*LRP5*) gene, located on chromosome 11q13, determined a HBM phenotype. Affected individuals in their series were asymptomatic and heterozygous for the mutation [25]. Notably in the same year, Boyden et al. described another series in which affected individuals carrying the same mutation had torus palatinus and facial deformities [33].

LRP5 and its cognate co-receptor low-density lipoprotein receptor-related protein 6 (LRP6) are single-pass transmembrane proteins with multiple domains. The fact that loss-of-function mutations in the *LRP5* gene affecting domains other than the first β-propeller are observed in osteoporosis-pseudoglioma syndrome (OMIM # 259770), an autosomal recessive condition causing early-onset and severe osteoporosis along with blindness [48], shows that this protein is a major regulator of bone formation. Another study revealed that common *LRP5* polymorphisms influence BMD in the general Caucasian population [49]. Indeed, LRP5 is a co-receptor for the canonical Wnt signaling pathway and is expressed in many cells, including osteoblasts. Physiologically, the binding of the first β-propeller domain of LRP5 co-receptor to Wnt determines the elevation of the intracellular concentration of β-catenin, which translocates to the nucleus and promotes the transcription of genes involved in bone formation [50]. The G171V mutation is thought to prevent the binding of the first β-propeller domain of LRP5 with sclerostin (SOST) and Dickkopf 1 (DKK1), which normally impair the binding of LRP5 with Wnt, with the net result of deficient inhibition of bone formation. Thus, the mutation in the first β-propeller domain of LRP5 results in the constitutive activation of the Wnt/β-catenin pathway and in increased bone formation [51], consistently with the phenotype seen in LRP5 HBM. Another hypothesis suggested that the G171V mutation disrupts the interaction between LRP5 and Mesd, a chaperone protein required for transport of the co-receptor to cell surface [52]. Mouse models with LRP5 G171V mutation are characterized by increased cortical thickness and area and increased ash weight and bone strength [38].

Exons 2, 3, and 4 of *LRP5* gene collectively code for the first β-propeller domain of the LRP5 protein. Several pathogenic missense mutations located in these exons other than G171V have been reported to reduce binding affinity with SOST and DKK1 through an analogous mechanism to that observed with the G171V mutation [36]. These include Q89R, R266C, and R154M in exon 2; N198S, N198Y, A214T, and W196G in exon 3; and M282V and A242T in exon 4. Furthermore, Roetzer et al. reported an in-frame duplication of 6 base pairs resulting in the insertion of two glycine residues [38]. Notably, one study recently reported the first missense mutation (R1414S) affecting a domain other than the first β-propeller domain of LRP5. The R1414S is in exon 20, which codes for the cytoplasmic domain of the LRP5 co-receptor protein; this affects the downstream release of β-catenin and thus can also determine HBM [42]. The different *LRP5* mutations and all the reports in which they were detected are listed in Table 2.

**Table 2:** The LRP5 mutations detected in cases of LRP5 HBM are reported. LRP5, low-density lipoprotein receptor-related protein 5. All mutations are missense except for one case of in-frame insertion of two residues (Roetzer et al., 2018 [38]). All mutations affect exons 2–4 except for LRP5 R1414S (Zhao et al., 2023 [42]), located in exon 20 coding for the cytoplasmic domain of the protein

Case report	Year	Mutation	Exon
Little et al. [25]	2002	LRP5 G171V	Exon 3
Boyden et al. [33]	2002	LRP5 G171V	Exon 3
Van Weesenbeck et al. [23]	2003	LRP5 A242T (referring to Beals et al. (2001) [46]) LRP5 A214V (referring to Renton et al. (2002) [21])	Exon 4 Exon 3
Boyden et al. [44]	2004	LRP5 G171V LRP5 N198S	Exon 3 Exon 3
Whyte MP et al. [43]	2004	LRP5 G171V	Exon 3
Kwee et al. [22]	2005	LRP5 A214T	Exon 3
Rickels et al. [34]	2005	LRP5 R154M	Exon 2
Balemans et al. [39]	2007	LRP5 M282V	Exon 4
Wang et al. [35]	2013	LRP5 A242T	Exon 4
Gregson et al. [36]	2016	LRP5 N198S LRP5 A242T LRP5 R266C LRP5 T173M LRP5 Q89R	Exon 3 Exon 4 Exon 2 Exon 3 Exon 2
Costantini et al. [37]	2017	LRP5 N198Y	Exon 3
Roetzer et al. [38]	2018	In-frame insertion of two glycines following residue 171 (p.G171_E172insGG)	Exon 3
Zhao et al. [42]	2023	LRP5 W196G LRP5 R1414S	Exon 3 Exon 20
Present paper	2023	LRP5 A242T	Exon 4

It is important to note that also mutations modifying the structure of the first β-propeller domain of LRP6 can be responsible for HBM phenotype. The first report on this was published in 2019 and described two families with gain-of-function mutations of the *LRP6* gene, thereby leading to the description of a new disorder, called LRP6 HBM, which is remarkably similar to LRP5 HBM, reflecting the cognate co-receptor function of LRP5 and LRP6 proteins. Indeed, when comparing the affected individuals with patients carrying *LRP5* mutations, no feature was found to differentiate LRP6 HBM from LRP5 HBM [3]. Up to date, four genetic defects affecting the exons coding for the first β-propeller domain of LRP6 have been identified [53].

Our patient carried the A242T mutation in exon 4 of *LRP5*, which has been predicted to destabilize the SOST binding site by disrupting the core packing of the LRP5 protein structure and by affecting the first β-propeller domain [36]. This mutation was detected in 2003 by Van Wesenbeeck et al. when examining individuals described elsewhere in previous publications, i.e., in one family from the US [41], one family from Italy [24], and one family from France in which the proband was diagnosed with “autosomal dominant osteopetrosis, type 1,” even though this was a mislabeling for LRP5 HBM as the described clinical and radiological findings were not typical for osteopetrosis. One family from China [35] and three families from the UK ([36] – Supporting data S5) also carried the A242T mutation. Concerning our patient, her sister and her children showed normal *DEXA* findings; since her parents were deceased at the time of LRP5 HBM diagnosis and the remaining relatives did not agree to undergo DNA testing, it was not possible to determine whether she carries a *de novo* mutation of the *LRP5* gene.

## Conclusions

LRP5 HBM is a rare, autosomal dominant endosteal hyperostosis for which no reviews have ever been published, perhaps due to the confusion resulting from the past use of several denominations for this condition especially before the identification of the role of the *LRP5* gene in the disease pathogenesis. For the first time, we aimed at characterizing this disorder with all the information available in the scientific literature by including also all the cases of Worth-type hyperostosis predating genetic analysis of LRP5, available since 2002. Although neurological complications have long been considered absent in the disease, we found that they are present in 19.4% of affected individuals. Elevated serum ALP levels are present in a minority of patients (3.7%), and facial changes and torus palatinus are not consistent findings, with a prevalence of approximately 61% and 41%, respectively. As previously mentioned, some subjects may be asymptomatic. Therefore, research of mutations of the *LRP5* gene is fundamental not only for diagnosis but also to differentiate LRP5 HBM from the more recently identified LRP6 HBM. In this paper, we reported the case of a 44-year-old woman affected by LRP5 HBM carrying the LRP5 A242T missense mutation. Facial dysmorphism, signs of intracranial hypertension, and multiple cranial nerve deficits were present; to our knowledge, our case is the first report of hypo-/anosmia in LRP5 HBM, suggesting a deficit of the first cranial nerve.
